# Vascular Smooth Muscle FTO Promotes Aortic Dissecting Aneurysms via m6A Modification of Klf5

**DOI:** 10.3389/fcvm.2020.592550

**Published:** 2020-11-20

**Authors:** Dong Ma, Xiao Liu, Jin-jin Zhang, Jun-jian Zhao, Yan-jie Xiong, Quan Chang, Hong-yan Wang, Peng Su, Jia Meng, Yong-bo Zhao

**Affiliations:** ^1^School of Public Health, North China University of Science and Technology, Tangshan, China; ^2^Cardiac Surgery Department, The Fourth Hospital of Hebei Medical University, Shijiazhuang, China; ^3^Affiliated Hospital of North China University of Technology, Tangshan, China

**Keywords:** aortic dissecting aneurysm, vascular smooth muscle cells, FTO, Klf5, GSK3β

## Abstract

**Background:** Aortic dissecting aneurysm (ADA) represents an aortic remodeling disease with a high mortality rate. Fat mass and obesity-associated protein (FTO) exerts RNA demethylation function to regulate gene expression related to stem cell differentiation, DNA damage repair, and tumorigenesis, but the role of FTO in ADA is still unclear.

**Methods:** The expression and location of FTO in 43 ADA tissues and 11 normal tissues were determined by RT-qPCR, WB, immunohistochemistry, and immunofluorescence staining. Detecting proliferation and migration of VSMCs. M6A methylated RNA immuno-precipitation qRT-PCR and dual luciferase reporter assay were performed for determining m6A level and interaction between m6A modulation and Klf5 mRNA, respectively.

**Results:** FTO are highly expressed in VSMCs. FTO was positively correlated with BMI, triglyceride, and D-dimer (all *P* < 0.05). Functionally, both AngII-induced FTO expression and over expression of FTO promote cell proliferation and migration, whereas knockdown of FTO inhibits these functions. Mechanically, we identified Krüppel-like factor 5 (Klf5) as a target of FTO mediating m6A modification. Overexpression of FTO reduced m6A modification on Klf5 mRNA and promoted Klf5 mRNA expression. Furthermore, the p-GSK3β and Klf5 levels increased after FTO overexpression. Finally, knockdown of FTO suppresses the p-GSK3β levels and Klf5 expression regardless of AngII treatment.

**Conclusions:** Our study revealed that FTO expression significantly contributes to the phenotype conversion of VSMCs and the ADA by the demethylation function (m6A), thereby providing a novel therapeutic target.

## Introduction

Aortic dissecting aneurysm (ADA) is linked to significant morbidity and mortality in the elder, characterized by a tear in the intimal layer of the aorta or bleeding within the aortic wall (1). Main risk factors leading to ADA include hypertension, dyslipidemia, and genetic disorders. At present, surgical treatment of ADA is the only strategy due to no effective drugs to prevent its formation and development (2). Highly phenotypically plastic as a key characteristic of vascular smooth muscle cells (VSMCs), its loss (from a contractile to a synthetic phenotype) has been recognized as an early event in ADA, which is verified in a multiple kinds of animal models and human ADA specimens (3). However, the underlying mechanisms of ADA formation and progress are still elusive.

Obesity as a main cardiovascular risk factor for atherothrombosis and aneurysms has been clearly demonstrated (4, 5). The fat and obesity-related (FTO) function as the m6A eraser of RNA modulation plays a critical oncogenes role in various types of tumors (6, 7), which belongs to the alkB family member of α-ketoglutarate-dependent dioxygenases (8). Studies have shown that mice lacking FTO exhibit severe growth retardation (9). Insufficient FTO gene or partial loss of FTO gene expression is related to a reduction of obesity, inversely, overexpression of FTO leads to obesity and weight gain in mice (10, 11). Recent researches have demonstrated that FTO acts as a key regulator in RNA demethylation function that can regulate gene expression and promote stem cell differentiation, DNA damage repair, and tumorigenesis, M6A demethylation by FTO affects mRNA expression and stability (12). Zhou S et al. found that FTO targets β-catenin through mRNA demethylation. Thereby, regulating chemoradiotherapy resistance in cervical squamous cell carcinoma (13). Recent studies reported that m6A modulation is involved in the proliferation and migration of VSMCs (14), the specific mechanism needs to be detailed yet.

Krüppel-like factor 5 (Klf5) is a zinc finger-containing transcriptional factor that regulates the expression of numerous genes involved in cardiovascular remodeling (15). The published research reported that it plays a critical role in the regulation of proliferation, extracellular matrix, migration, and inflammation in VSMCs, all of which are essential to cardiovascular diseases (16, 17). Our previous study also showed that the inhibition of Klf5 expression in macrophages alleviated abdominal aortic aneurysm (18). However, its distinctive contribution to VSMC during ADA procession has not been investigated.

The purpose of this study was to investigate the role of FTO in ADA and detect whether FTO affects the expression of Klf5 in VSMCs via the demethylation function of FTO. We found that the activation of FTO-associated Klf5 expression and signaling pathway was involved in the phenotypic modulation of VSMCs and in the early and advanced ADA.

## Materials and Methods

### Clinical Specimens

Forty-three cases of ADA patients' tissue (including 29 males and 14 females, aged from 22 to 74 years) were harvested from January 2018 to June 2019 in the Forth Hospital of Hebei Medical University. They were divided into the AD group (aortic dissection, AD, *n* = 31, including standard type A 25 cases and type B six cases) and AA group (aortic aneurysm, AA, *n* = 12, thoracic aortic aneurysm four cases and abdominal aortic aneurysm eight cases). Normal aortic tissue from post-mortem subjects were made available representing controls (*n* = 11). Consent gained from ADA patients after surgery for the use of aortic tissue in research. Inclusion criteria include the complete demographic characteristics and axial computed tomography scans for demonstration of AD and AA, and exclusion criteria is Marfan syndrome and missing clinical data for ADA patients. Collection of clinical specimens ensures compliance with the regulations of the fourth Hospital of Hebei Medical University.

### Histology

Vascular tissue were sliced for 4 μm and dewaxed, and then hematoxylin eosin (HE) staining, dehydrated in 70–100% ethanol, immersion in xylene solution, sealed in neutral gum, microscopy observation.

### Cell Culture and Transfection

Human aortic vascular smooth muscle cells (VSMCs) (ScienCell, no. 6,110) were routinely cultured as previously described (17). Eighty percentage confluence for cultured cells were passaged. FTO siRNA, Klf5 siRNA, and corresponding control (GenePharma, Shanghai, China) were transfected into VSMCs by the Lipofectamine 2000 (Thermo Fisher Scientific, Waltham, MA, USA) as well as Lentivirus-FTO and GFP controls (Hanbio, Shanghai, China) infection compliance with the manufacturer's protocols. FTO mutation (including H231A and D233A point mutations, which disrupt the enzymatic activity) was conducted according to references (19, 20).

### RNA Extraction and RT-qPCR RNA

TRizol reagent and reverse transcriptase kit (Invitrogen) was used for the extraction of total RNA and synthesis of cDNA, respectively. Real-time quantitive PCR (qRT-PCR) was performed for relative mRNA expression using the 2^Δ*ΔCt*^ formula as previously described (15). The primer sequences of human FTO and control GAPDH are as follows:

FTO-F: 5′-CTGGTTTGGCGATACCCCTT-3′; FTO-R: 5′-CAGCCACTCAAACTCGACCT-3; Klf5-F:

5′-GGACTCATACGGGCGAGAAG-3′; Klf5-R: 5′- TAAAGGATGGCAGAGCGGAC−3′; GAPDH-F:

5′-GGGTGTGAACCATGAGAAGTATGAC-3′; GAPDH-R: 5′-GTGGTCATGAGTCCTTCCACGATACC3′.

### Western Blotting Assay

Western blot was performed as previously described (17). Primary antibodies include Klf5 antibody (GTX103289, GeneTex, BD Biosciences), anti-N6-methyladenosine (m6A) (ab151230, Abcam), β-actin (ab8226, Abcam), and antibodies of FTO (27226-1-AP), PCNA (10205-2-AP), p-GSK3β (14850-1-AP), GSK3β (24198-1-AP), p-AKT (66444-1-AP), AKT (10176-2-AP), p-ERK (20582-1-Ig), ERK (16443-1-AP) were from Proteintech (USA). The Image J software (NIH) was used for the quantification of band intensities.

### Immunohistochemical Staining

Immunohistochemical staining was performed by the SP method as previously described (17). Primary anti-human polyclonal antibody FTO. The percentage of brown particles in five random medium-film fields was quantified by the IPP 6.0 image analysis software.

### Immunofluorescence Staining

Immunofluorescence staining was performed as previously described (15). Primary rabbit anti-human polyclonal antibody FTO (1:50 dilution) and mouse anti-human polyclonal antibody α-actin (1:100 dilution) were incubated overnight at four. Secondary antibodies (1:200; 021516, and 031806, KPL, USA) were incubated at room temperature for 45 min. DAPI for cell nuclear staining (157574, MB Biomedical). Image J software was used for analysis of the fluorescence intensity of images from laser confocal microscope (magnification 630).

### MTT Assay

Colorimetric assay for VSMC viability was examined by MTT [3-(4,5-dimethylthiazol-2-yl)-2,5-diphenyltetrazolium bromide] (Sigma-Aldrich, St. Louis, MO, USA) as previously described (6), and the absorbance was measured at 450 nm using a microplate reader (Termo Fisher, USA).

### Scratch Assay

Scratch wound assay for treated VSMCs as previously described (17). Microscope (OLYMPUS, CKX41) equipped with a camera (Canon, EOS 600D) was used for images.

### Measurement of Total m6A Level

Total mRNA m6A levels were detected by the m6A RNA methylation quantification ELISA kit (Epigentek). PolyA^+^ mRNA was purified using the GenElute mRNA Mini-prep Kit (Sigma-Aldrich) according to the manufacturer's instructions. 200 ng mRNA was used per well (6).

### m6A-RNA Immunoprecipitation (MeRIP) Assay

MeRIP-qPCR analysis was used as a reported method (6). Briefly, the total RNAs were first extracted from treating VSMCs (ThermoFisher). The RNA concentration was adjusted to 1 μg/μl. RNA was fragmented into ~100 nt size and these RNA were immunoprecipitated with the anti-m6A antibody according to the standard protocol of the Magna MeRIP m6A Kit (Merck Millipore). m6A enrichment was determined by qPCR analysis. Primers targeting m6A enriched regions of Klf5 (two sites are 713 and 1,457) are available in following. Klf5-713-F: 5′-CCTTCGTGAGCGTCTGGCTGCC-3′; Klf5-713-R: 5′-CGTGTTTCAGATCGTCTCCGGAGAAGAG-3′; Klf5-1,457-F: 5′-GTCGTCTCACTTAAAAGCTCACCTGAGG-3′; Klf5-1457-R: 5′-CCGTGTGCTTCCTGTAGTGGCGG-3′; All data were analyzed by adopting 2^−Δ*ΔCt*^ methods.

### Luciferase Reporter Assay

Luciferase vectors (Promega) were used for reconstructed plasmid with a firefly luciferase (F-luc) and a renilla luciferase (R-luc). Wild-type Klf5 reporter plasmid was cloned by inserting the full-length of Klf5 transcript after the F-luc coding sequence. The mutant Klf5 reporter plasmid was replaced adenosine bases within the m6A consensus sequences with cytosine. Wild-type and mutated F-luc-Klf5 plasmids (500 ng) were transfected into HEK293 cells in 6-well for 48 h. F-luc activity was assayed by the dual-glo luciferase system (Promega) to evaluate the effect of m6A site on Klf5. R-luc was used as a normalize the transfection efficiency.

### Statistical Analysis

Measurement data were presented as mean ± standard deviation. Student's *t* test and one-way analysis of variance (ANOVA) were applied to analyze the comparisons between two groups and among the multiple groups under the SPSS 23.0 software, respectively. Count data were tested by the Chi-square (χ^2^) test or Fisher's exact test. The routine distribution of variables for each group is tested. If it is non-normal, you need to apply the variable transformation to normalize it and then analyze it further. *P* < 0.05 was considered statistically significant.

## Results

### FTO is Highly Expressed in Human ADA Tissues

**Figure 1:**
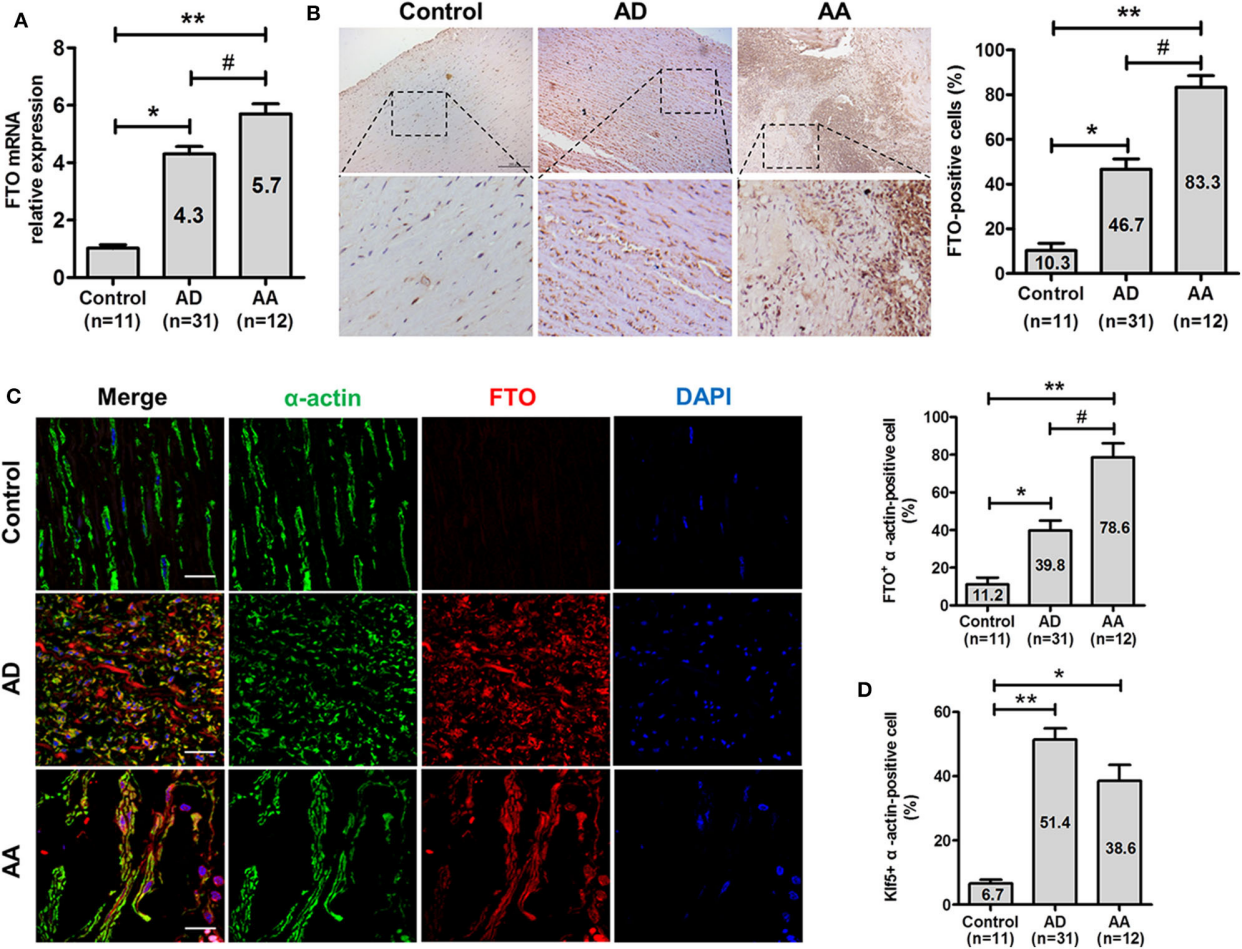
FTO is highly expressed in human aortic dissection (AD) and aortic aneurysm (AA). **(A)** qRT-PCR for FTO expression in human AD (*n* = 31), AA tissues (*n* = 12), and control (*n* = 11). **P < 0.01 vs. Control, ^#^*P* < 0.05 vs. AD. **(B)** Representative photographs of immunohistochemical staining for FTO in human ADA tissues and control. Scale bar = 100 μm. Right: Statistics of FTO -positive cells in human ADA, RADA tissue, and control. *P < 0.05, **P < 0.01 vs. control, ^#^*P* < 0.05 vs. ADA. **(C)** Confocal immunofluorescence for human ADA and control sections stained with α-actin, FTO, and 4′,6-diamidino-2-phenylindole (DAPI). Right: statistics of FTO^+^ α-actin–positive cells in human AD, AA tissues, and control. **P < 0.01 vs. Control, #P < 0.05 vs. ADA. **(D)** Statistics of Klf5^+^ α-actin–positive cells in human AD, AA tissues and control. All data were present mean ± SD, *P < 0.05 and ^**^P < 0.01 vs. Control.

To investigate the potential relevance of FTO in human ADA, qRT-PCR and immunohistochemical analysis were performed on AD, AA, and control aortas. As shown in Figures 1A,B, FTO mRNA expressions were significantly higher (ADA: 4.3-fold and RADA: 5.7-fold vs. Control, *P* < 0.05, and *P* < 0.01, respectively) in human ADA and RADA than in control (Figure 1A). Likewise, FTO immunostaining were consistently increased in human AD and AA tissues compared with that in control, and the percentages of FTO-positive cells were significantly higher in AD and AA than that in control (AD: 46.7 ± 8.0%, AA: 83.3 ± 9.1% vs. Control: 10.3 ± 5.5%, *P* < 0.05, and *P* < 0.01, respectively). Furthermore, we identify vascular smooth muscle cells (VSMCs) expressing FTO in human AD and AA tissues using confocal fluorescence microscopy, as showed in Figure 1C. The results demonstrated that the increased FTO expression was primarily localized to intramural VSMCs (AD: 39.8 ± 5.2%, AA: 78.6 ± 7.4% vs. Control: 11.2 ± 3.6%, *P* < 0.05, and *P* < 0.01, respectively). Parallelly, Klf5 expression was also significantly higher in AD and AA tissues than control (AD: 51.4 ± 3.5%, AA: 38.6 ± 4.9% vs. Control: 6.7 ± 1.1%, *P* < 0.01, and *P* < 0.05, respectively) (Figure 1D).

In addition, correlation analysis of FTO expression in vascular tissues of ADA with clinicopathological parameters showed that FTO expression has no correlation with gender, age, smoking history, and total cholesterol (*P* > 0.05), however, FTO expression is positively associated with BMI, triglyceride, and D-dimer (*P* < 0.01) (Table 1). These data suggest that FTO and Klf5 may be relevant to the formation and progress of human ADA.

**Table 1:** Relationship between FTO expression and clinicopathological features in ADA patients.

**Clinicopathological characteristics**	***n***	**FTO High-expression**	**FTO Low-expression**	**χ^2^**	***P***
**Gender**				0.188	0.485
Male	29	21	8		
Female	14	11	3		
**BMI**				12.683	0.002
Low weight Normal range Overweight	8 13 22	1 6 18	7 7 4		
**Age**				1.418	0.234
<51 ≥51	18 25	9 17	9 8		
**Hypertension**				0.525	0.469
Yes No	28 15	21	7 5		
**Smoking history**				0.312	0.576
Yes No	25 18	16 10	9 8		
**Triglyceride**				6.661	0.012
Normal range Rise	20 23	9 19	11 4		
**Total cholesterol**				0.290	0.590
Normal range Rise	33 10	20 7	13 3		
**D-dimer**				4.081	0.043
Normal range Rise	11 32	6 27	5 5		

### FTO Expression Promotes VSMC Proliferation and Migration

**Figure 2:**
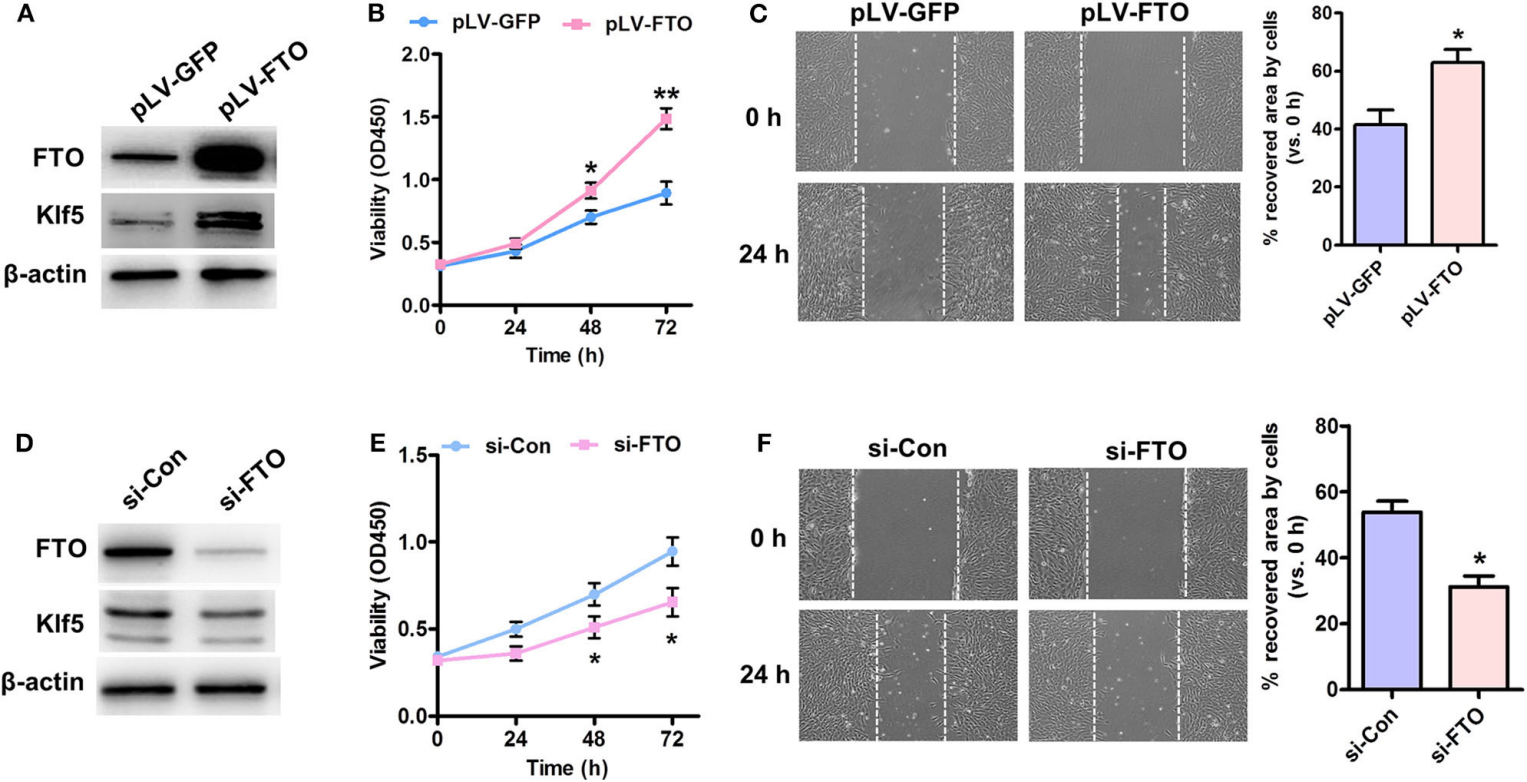
Effect of Forced expression or knockdown of FTO on proliferation and migration of VSMCs. **(A)** Human VSMCs were infected with Lentivirus encoding FTO (pLV-FTO) or pLV-GFP for 48 h, and then western blot detected the expressions of FTO and Klf5. **(B)** MTT assay for cell proliferation in pLV-FTO-infected VSMCs. *P < 0.05, **P < 0.01 vs. pLV-GFP at the corresponding time point. **(C)** A scratch assay for the pLV-GFP- or pLV-FTO-infected VSMCs at 24 h later. Right: statistic of migrating cells in the scratch gap. *n* = 3. *P < 0.05 vs. pLV-GFP. **(D)** VSMCs were transfected with siRNA against FTO (si-FTO) or non-specific siRNA (si-Con) for 24 h, and then the expressions of FTO and Klf5 were examined by western blot. **(E)** MTT assay for effects of FTO knockdown on cell proliferation. *P < 0.05 vs. si-Con at the corresponding time point. **(F)** Scratch assay for the si-Con or si-FTO-transfected VSMCs at 24 h later. Right: statistic of migrating cells in the scratch gap. The data present mean ± SEM from three independent experiments. *P < 0.05 vs. si-Con.

Up-regulation of KLF5 is implicated in vascular remodeling and lipid metabolism (21, 22), FTO was overexpressed or knockdown in human VSMCs to address its role in cell proliferation and migration as well as to clarify the relationship between FTO and Klf5. As showed in Figure 2A, pLV-FTO treatment significantly increased cellular FTO and Klf5 protein levels. The mTT assay showed that pLV-FTO-infected cells exhibited higher cell viability than pLV-GFP-treated cells (Figure 2B). And wound-scratch healing assay further verified pLV-FTO-infected cells exhibiting higher cell migration (Figure 2C). Conversely, siRNA-mediated FTO silencing reduced the expressions of FTO and Klf5 (Figure 2D), along with the inhibition of proliferation (Figure 2E) and migration (Figure 2F). The results suggest that FTO-induced VSMC proliferation and migration are related to KLF5 expression.

### FTO-Mediated Ang II-Induced VSMC Proliferation and Migration

**Figure 3:**
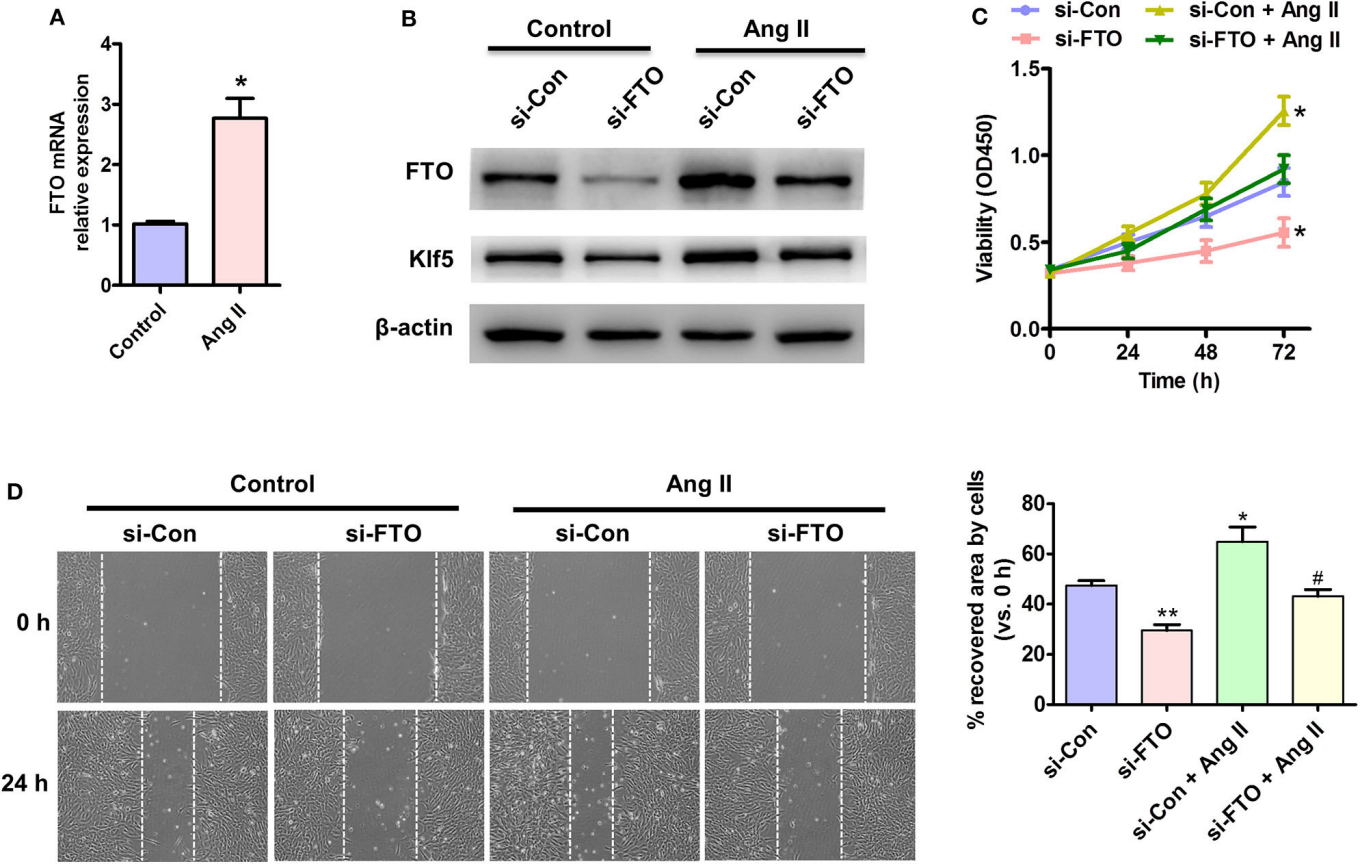
Effects of FTO on Ang II-induced VSMC proliferation and migration. **(A)** qRT-PCR detection for mRNA level of FTO in Ang II (100 nmol/L)-treated human VSMCs for 12 h. *P < 0.05 vs. Control. **(B)** VSMCs were transfected with si-FTO or si-Con RNA for 24 h and consequent with AngII treatment for 24 h. Western blotting assay for expression of FTO and Klf5. β-actin was used as a loading control. **(C)** MTT assay for proliferation of treated cell at different time points. **(D)** VSMCs were treated as **(B)** and scratch assay for cell migration. Right: statistic of migrating cells in the scratch gap. Data present mean ± SEM, n = 3. *P < 0.05 and **P < 0.01 vs. si-Con. ^**#**^*P* < 0.05 vs. si-Con + AngII.

Because loss of endothelial FTO prevents the progress of obesity-induced hypertension (23), and KLF5 is an essential regulator of angiotensin II signaling-associated cardiovascular remodeling (21), we attempted to elucidate the underlying mechanisms by which both FTO and Klf5-mediated angiotensin II (AngII) induced proliferation and migration of VSMCs. AngII-treated VSMCs significantly up-regulated the expression of FTO at transcription and protein levels (Figures 3A,B). Importantly, inhibition of FTO expression by si-FTO transfection reduced AngII-induced Klf5 expression (Figure 3B), which plays an essential role on AngII-induced VSMC proliferation (24, 25). Correspondingly, MTT and wound-healing assays indicated that AngII promoted VSMC proliferation and migration, whereas repressing the expression of FTO inhibited the AngII-induced VSMC proliferation (Figure 3C) and migration (Figure 3D), further suggesting that FTO may exert the effects of VSMC proliferation and migration via Klf5 expression.

### FTO-Mediated mRNA Expression of Krüppel-like Factor 5 (KLF5) Depends on Its m6A Demethylase Activity

**Figure 4:**
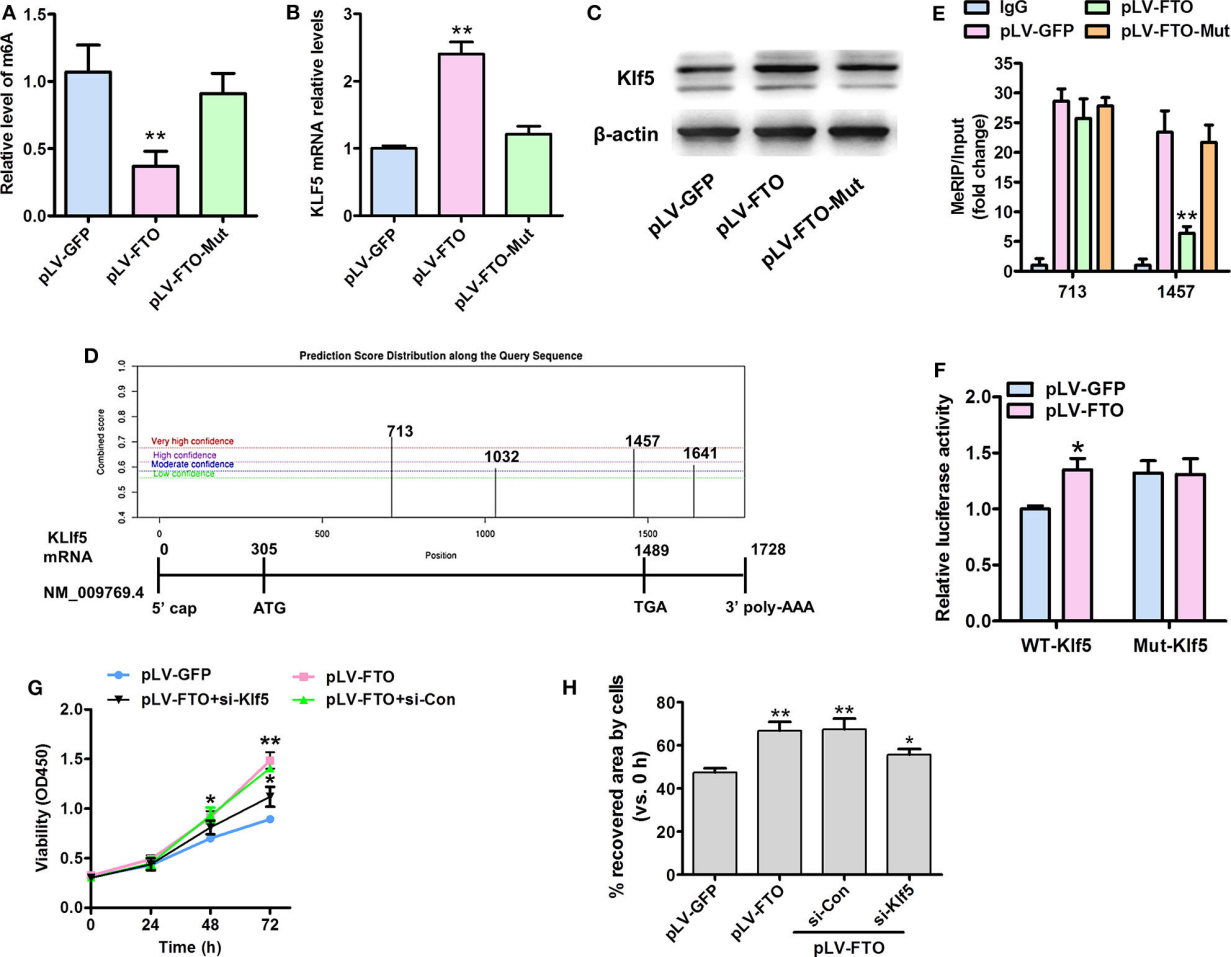
FTO-mediated mRNA expression of Klf5 depends on its m6A demethylase activity. **(A)** A sketch map of 0 to +1,728 bp region of the Klf5 mRNA (NM:009769.4) showing the position of 4 KLF5-m6A sites. **(B)** ELISA assays of total m6A levels in human VSMCs infected with pLV-FTO, FTO mutant (H231A and D233A; pLV-FTO-Mut) or pLV-GFP. **(C,D)** qRT-PCR **(C)** and Western blot assay **(D)** for Klf5 mRNA and protein expressions, respectively. **P < 0.01 vs. pLV-GFP. **(E)** Methylated RNA immunoprecipitation (MeRIP)-qPCR analysis of FTO-mediated m6A modifications on Klf5 mRNA in VSMCs infected with the pLV-FTO, pLV-FTO-Mut, or pLV-GFP. **(F)** Wild-type (WT-Klf5) or m6A consensus sequence mutant Klf5 cDNA (Mut-Klf5) was reconstructed with firefly luciferase reporter. Relative luciferase activity was measured in WT-Klf5 and Mut-Klf5 (A-to-C mutation on m6A site 1,457) after co-infection with pLV-FTO or pLV-GFP in HEK293 cell. Data were mean ± SD, *n* = 3. *P < 0.05, **P <0.01 vs. pLV-GFP. **(G)** VSMCs infected with pLV-GFP or pLV-FTO, and then transfected with si-Con or si-Klf5 for 24 h, MTT assay for the treated cell at corresponding time points. *P < 0.05 and **P < 0.01 vs. pLV-GFP group. **(H)** Wound-scratch healing assay for the treated cell migration at 24 h. *P < 0.05 and **P < 0.01 vs. pLV-GFP.

It remains unknown whether demethylase FTO regulates Klf5 expression dependent on the demethylation activity. As expected, pLV-FTO (lentivirally translated wild-type FTO), but not the pLV-FTO-Mut remarkably decreased the global mRNA m6A level (Figure 4A) and increased Klf5 expression at transcription (Figure 4B) and protein levels (Figure 4C) in treated VSMCs. To define the molecular mechanism by which demethylase FTO affected Klf5 expression, we utilized the m6A site public database (m6AVars) (http://m6avar. Renlab.org) and identified four potential m6A modification sites in the 0 to +1,728 be a region of the Klf5 mRNA (Figure 4D), and we validated two high score of m6A modification sites (sites 713 and 1,457) using the MeRIP-qPCR method. Moreover, pLV-FTO dramatically reduced the m6A level of Klf5 mRNA compared with pLV-FTO-Mut or pLV-GFP at 1,457 sites, but no effects on the site 713 (Figure 4E). Subsequently, we constructed both wild-type and mutant Klf5 reporter plasmids (mutation A to C of 1,457 site) to further prove the effect of m6A modification (1,457 site) on Klf5 expression. As showed in Figure 4F, luciferase activity of the WT-Klf5 reporter was significantly enhanced upon pLV-FTO infection, whereas it didn't affect the luciferase activity of the Mut-Klf5 reporter, suggesting that Klf5 is functionally an important target of FTO via its m6A demethylase activity in the Klf5 mRNA transcript. In addition, reduced KLF5 expression by transacting with siRNA against KLF5 in pLV-FTO-infected VSMCs partly suppresses the pro-proliferation (Figure 4G) and pro-migration (Figure 4H) effectiveness of FTO overexpression. These findings suggest that FTO regulates Klf5 expression by the m6A modulation of Klf5 mRNA.

### FTO Regulates Klf5 Expression and Enhances GSK3β Signal Pathway in VSMCs

**Figure 5:**
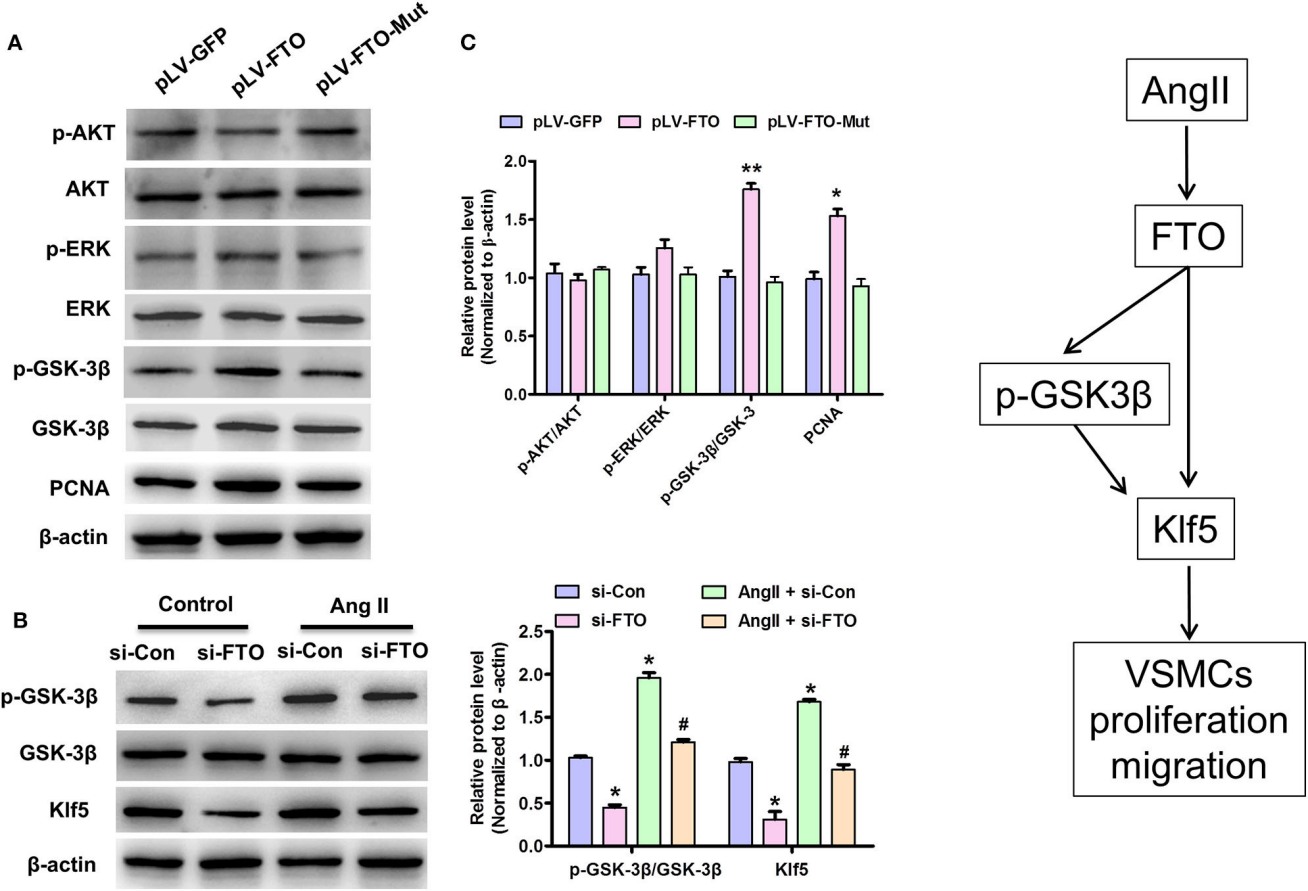
FTO regulates Klf5 expression and enhances GSK3β signal pathway in VSMCs. **(A)** VSMCs infected with pLV-GFP pLV-FTO or pLV-FTO-Mut and then western blotting assay for detection of the protein levels of AKT, p-AKT, ERK, p-ERK, GSK-3β, p-GSK-3β, and PCNA. Right: statistic of band intensities were shown below (*n* = 3). *P < 0.05, **P < 0.01 vs. pLV-GFP. **(B)** VSMCs were transfected with si-FTO or si-Con and then treated with AngII for 24 h. *P < 0.05 and **P < 0.01 vs. si-Con. ^**#**^*P* < 0.05 vs. si-Con + AngII. **(C)** A working model for FTO-mediated Klf5 expression promoting VSMC proliferation and migration via decrease in the m6A modulation of Klf5 mRNA and p-GSK-3β level.

Because activation of AKT and EKR signaling is required for VSMC proliferation and migration (26) as well as GSK3β mediates Klf5 degradation, whereas phosphorylated-GSK3β up-regulates Klf5 phosphorylation modification and then entrancing nuclear for regulating gene expression related to proliferation and migration (26), we performed further experiments using FTO and mut-FTO-overexpressing human VSMCs to investigate expression and m6A demethylase activity of FTO whether affects these signaling pathways. In these experiments, overexpression of WT-FTO, but not Mut-FTO significantly increased p-GSK3β expression, while increasing PCNA expression (Figure 5A). And, enhanced expression of WT-FTO only slightly unregulated p-ERK expression and did not affect the AKT signal (Figure 5A). In addition, we also observed inactivation of GSK3β signaling in AngII-treated VSMCs, however, knockdown of FTO by si-FTO partly reversed AngII-induced inactivation of GSK3β signaling and inhibited Klf5 expression (Figure 5B). These data indicate that FTO upregulates Klf5 expression by the inactivation of GSK3β signaling pathway.

## Discussion

This study proves that FTO has a vital effect on proliferation and migration of VSMCs via regulating Klf5 expression by m6A modulation on its mRNA and GSK-3β signaling pathway (Figure 5C). Remarkably, we observed that the elevated FTO expression in human ADA tissues is positively associated with BMI, triglyceride, and D-dimer (*P* < 0.01) (Table 1). Forced expression of FTO promotes the proliferation and migration of VSMCs and up-regulation of Klf5 expression; conversely, knockdown of FTO suppresses Klf5 expression and cell proliferation and migration regardless of AngII stimulation. As m6A RNA demethylase, FTO-mediated Klf5 expression dependent on its m6A demethylase activity as mutations in the FTO catalytic domain strongly decrease Klf5 expression at mRNA and protein levels. Our m6A modulation prediction and detection on Klf5 mRNA as well as the subsequent luciferase reporter/mutagenesis assay validation suggest that Klf5 is a critical target gene of FTO. Meanwhile, FTO expression is also involved in AngII-induced activation of GSK-3β signaling pathway, which regulates Klf5 expression as previous studies (27). A schematic model summarizing our discoveries is shown in Figure 5C.

Although obesity, hypertension, and genetics have been reported as main risk factors associated with ADA (28–30) as well as the strong correlation between FTO gene and obesity and diabetes has been confirmed (23, 31), in regards to the role of FTO in formation and progress of ADA remains unclear. In this study, elevated FTO in ADA tissues is positively associated with the clinical parameters (BMI, plasma triglyceride, and D-dimer) of ADA patients, and previous studies demonstrated that the biochemical indicators triglyceride and D-dimer were positively correlated with the incidence of the ADA (32), demonstrating that FTO may be involved in the formation and progress of the ADA.

The VSMC is the principal cell that constitutes the aortic layer, the triggers including platelet-derived growth factor, angiotensin II, inflammatory cytokines, and other stimuli lead to its phenotypic transformation in almost all types of ADA (33, 34). Targeting the key regulators controlling the phenotype modulation of VSMCs could be maintaining vascular homeostasis and suppressing ADA (35–37). In this study, we found that FTO up-regulation promotes shift from contractile to proliferating phenotype in VSMCs, which is similar to its oncogenic roles (7, 38). Moreover, these roles rely on regulating Klf5 expression, an important regulator of vascular remodeling in AngII signaling pathway (15). Thus, intervention of expression or activity of FTO may be a novel strategy for ADA treatment.

N6-Methyladenosine (m6A) is a common epigenetic modification in RNAs contributing to tissue development, stem cell self-renewal and differentiation, as well as mRNA stability, splicing, transport, localization, translation, and so on (15, 39, 40). In spite of FTO being able to regulate fat metabolism and reduce m6A levels, its ability to regulate vascular remodeling by its m6A demethylase activity is still unknown. Our results firstly suggest that alternative m6A modulation in 1,457 site of FTO target Klf5 mRNA promote their stability. Of note, 1,457 site in 3′ UTRs and near stop codons of Klf5 mRNA means that FTO demethylation is associated with upregulation of Klf5 expression according to previous reports (41). Under FTO overexpression or knockdown in VSMCs, we also found that increased or decreased Klf5 also promotes or suppresses cell proliferation and migration, respectively.

In addition, we also found that increased FTO led to GSK-3β phosphorylation at S9, which inactivates GSK3β (42). Since GSK-3β has been demonstrated to directly phosphorylate KLF5 at S303 and then targets it for ubiquitination and degradation (43), suggesting that FTO upregulates Klf5 expression at protein level via inactivation of GSK-3β inhibits its phosphorylation and degradation. Additionally, previous reports show that PI3K/AKT signaling pathway mediates FTO-induced the energy metabolism of breast cancer cell (44), and proliferation-related ERK signaling pathway is activated heavily in aneurysms (45), however, in this study, we found that overexpression of FTO has little effects on these two signaling pathways in VSMCs. These findings indicate that FTO correlates with the distinct signaling pathways in the different cell contexts and particular pathways to exert corresponding functions.

Despite the fact that the up-regulation of FTO in VSMCs is associated with ADA, some limitations should be attention in this study: First, the conclusion of this study needs to be validated in more human specimens. Second, verification of FTO-associated the pathological mechanism of ADA and regulation of Klf5 expression *in vivo* is necessary, which is our next work to prove it.

In conclusion, AngII induces FTO up-regulation mediating VSMC phenotypic switching via regulation of m6A demethylation on Klf5 mRNA and inactivation of GSK3β signaling pathway, suggesting that vascular smooth muscle FTO could be a novel therapeutic target for the prevention of ADA-associated diseases.

## Data Availability Statement

The raw data supporting the conclusions of this article will be made available by the authors, without undue reservation.

## Ethics Statement

Written informed consent was obtained from Ethics Committee of North China University of Science and Technology (2020316) for the publication of any potentially identifiable images or data included in this article.

## Author Contributions

Y-bZ, XL, J-jZhan, H-yW, and PS: collection and analysis of clinical data. Y-bZ: wrote the first draft of the article. J-jZhan, H-yW, PS, JM, J-jZhao, and Y-jX: design and completion of experiment. DM: critical revision. All authors have read and approved the manuscript.

## Conflict of Interest

The authors declare that the research was conducted in the absence of any commercial or financial relationships that could be construed as a potential conflict of interest.
